# Automatic and Focused Hair Pulling in Trichotillomania: Valid and Useful Subtypes?

**DOI:** 10.1016/j.psychres.2021.114269

**Published:** 2021-11-02

**Authors:** Jon E. Grant, Samuel R. Chamberlain

**Affiliations:** a Department of Psychiatry and Behavioral Neuroscience, University of Chicago, Chicago, IL USA; b Department of Psychiatry, Faculty of Medicine, University of Southampton, Southampton, UK; and Southern Health NHS Foundation Trust, Southampton, UK

**Keywords:** trichotillomania, automatic, focused, subtypes, comorbidity

## Abstract

Prior work suggested that trichotillomania may have four subtypes based on the extent to which pulling is automatic or focused in nature. 238 adults with trichotillomania undertook clinical and cognitive assessments and were assigned into four subtypes based on k-means clustering of *Milwaukee Inventory for Subtypes of Trichotillomania-Adult Version (MIST-A)* scores. We examined whether a cluster solution was apparent using conventional metrics. Based on prior literature, we then force-fitted a four subtype model (low-low, low-high, high-low, high-high). Subtypes were compared and validity of the MIST-A subtyping approach was evaluated. A cluster solution did not converge based on conventional metrics. Following force-fitting, subtypes did not differ on demographic variables, age at symptom onset, nor duration of illness. The high-focused high-automatic subtype had worse symptom severity than other subtypes. Co-morbid depression was more common in the low-focused low-automatic and high-focused low-automatic subtypes. This study suggests that MIST-A subtypes may not be valid or clinically useful based on several issues. First, k-means models indicated that the MIST-A data did not generate any cluster solutions. Second, when a forced cluster solution was fitted, the subtypes did not differ on the vast majority of measures. Third, force-fitting four subtypes yielded findings that were logically inconsistent (e.g. worse quality of life in one group, but higher rates of comorbid anxiety/depression in others). Overall, we suggest that both focused and automatic pulling may characterize the same pulling episode, or certainly the same person across episodes. Thus they may be clinically relevant variables, but not forming coherent subtypes.

## 1 Introduction

Trichotillomania is characterized by the repetitive pulling out of one’s own hair resulting in distress and/or functional impairment (Grant and Chamberlain, 2016). Trichotillomania has defied any easy understanding, and as a consequence, has been difficult to treat with currently available options (Rothbart et al., 2013; Farhat et al., 2020). One common thought is that perhaps there are subtypes of trichotillomania and by understanding those subtypes, we could target treatments more effectively. Many theories of how to assess these subtypes have been suggested and have included gender, age of onset, family history, and comorbidity (Redden et al., 2016; Rickets et al., 2019; Lochner et al., 2019; Grant et al., 2021). Another school of thought focuses on differences in the underlying psychological mechanisms driving the hair pulling (Christenson et al., 1993). Toward that end, many people report not being fully aware of their pulling (referred to as “automatic” pulling), whereas “focused” pulling generally occurs when the person has awareness of pulling and intends to pull (e.g., remove a kinky hair that feels “not right”) (Christenson et al., 1991; du Toit et al., 2001). Could style of pulling therefore be useful in better understanding this behavior?

In an online survey of 1545 adults, Flessner and colleagues (2008a) found that adults with trichotillomania who scored high on either a scale of automatic pulling or focused pulling reported more severe trichotillomania symptoms and greater functional impact (using the Milwaukee Inventory for Subtypes of Trichotillomania-Adult Version [MIST-A] and a median-split procedure). Thus, the authors proposed four pulling subtypes or styles: low automatic-low focused, low automatic-high focused, high automatic-low focused, and high automatic-high focused. A study by Tung and colleagues in 187 adults with trichotillomania further found significant correlations between focused pulling and poor quality of life, which appeared to be largely statistically mediated by depression (2014). A large study of adults with trichotillomania who were seen in person across four sites (n=279) found three subtypes of trichotillomania, using a data-driven approach (mixture modelling statistics) across a range of measures (not only automatic vs focused). One subtype was characterized by highly focused pulling, infrequent and low intensity urges to pull, and low frequency of pulling behavior whereas the most common subtype reported high automatic pulling, with more pulling due to emotional triggers (Grant et al., 2021). In the case of children, Flessner and colleagues similarly examined a group of 186 youth via the same online platform and found that “high-focused” pullers reported more severe trichotillomania than “low-focused” pullers (Flessner et al., 2008b). There is also some indication that youth with trichotillomania develop more focused pulling as they get older (Panza et al., 2013) and that greater focused pulling among children may be associated with poorer long-term prognosis (Schumer et al., 2015). Interestingly, a recent study of 40 youth with trichotillomania demonstrated that focused pulling style largely improved with habit reversal therapy but there was limited improvement for automatic pulling (McGuire et al., 2020).

Of course the concepts of automatic and focused pulling are not absolute. People often report both focused and automatic pulling within the same pulling episode, or that they fluctuate between more focused or more automatic pulling over the course of their illness (Christenson et al., 1991, 1993). In fact, the very terms “automatic” and “focused” may not be perfectly defined for researchers or for the people with trichotillomania. Should these terms be referencing the amount of pulling time, the frequency of the desire to pull, or the intensity of the desire to pull? A recent study found that one possible subtype of trichotillomania was characterized by high focused pulling and yet they reported low urge intensity to pull (Grant et al., 2021). This could mean that higher focused pulling is more about frequency of having conscious desires to pull but that the intensity of the focused urge may not be high. Are these seeming contradictions inherent in the scales or do they reflect the dynamic nature of pulling for many people with trichotillomania?

Previous studies suggest that despite the limitations of the scales that understanding automatic and focused pulling may be useful in developing a greater understanding of trichotillomania. The results, however, seem to conflict with some research supporting the idea that more focused pulling leads to more severe symptoms but other studies suggesting not unless depression also co-occurs. Also, studies tended to assume the validity of these subtypes rather than testing their validity statistically. Thus, what does one make of these findings and are they useful clinically? In light of the current mixed findings in the literature, we sought to clarify the relationship of symptom severity and style of pulling and hypothesized that there would be a difference in symptom severity and co-occurring disorders in those with more focused pulling.

## 2 Methods

### 2.1 Participants

Adults, ages 18-65 with a primary and current DSM-5 diagnosis of trichotillomania were enrolled for various research studies over the period from 2009 to 2021 (all who met criteria under DSM-IV were reevaluated using DSM-5 criteria). Participants were primarily recruited from a large urban area using referrals and advertisements (online and print). Exclusion criteria included: (1) any neurological or psychiatric conditions that would prohibit completion of questionnaires, and (2) inconsistent or unstable psychotropic medication use or psychotherapy participation within the prior three months.

Data were collected at the University of Chicago following Institutional Review Board approval of the studies and associated consent procedures. Participants were given a comprehensive explanation of the study procedures and were given the opportunity to put forth any questions. After all questions were answered, participants provided written informed consent. This research was carried out in accordance with the principles of the Declaration of Helsinki.

### 2.2 Assessments

Participants were diagnosed using DSM criteria using a structured Clinical Instrument module (Minnesota Impulse Disorders Inventory, MIDI) (Grant, 2008; Chamberlain and Grant, 2018). Baseline demographic information included current age, self-identified race-ethnic grouping, educational background, employment, and marital status. Clinical characteristics were assessed with a semi-structured interview including questions regarding hair-pulling behavior. All participants were screened for co-occurring or lifetime psychiatric disorders using the Mini-International Neuropsychiatric Interview (MINI) (Sheehan et al., 1998).

In addition, the following clinical measures were used to assess trichotillomania symptom severity, types of pulling, and quality of life (all measures have previously demonstrated excellent psychometric properties in studies of trichotillomania): *Massachusetts General Hospital Hair-Pulling Scale (MGH-HPS)* is a seven-item Likert-type scale used to assess severity of hair-pulling in several different domains (Keuthen et al., 2005); *Milwaukee Inventory for Subtypes of Trichotillomania-Adult Version (MIST-A)* (Flessner et al., 2008a); and the *Quality of Life Inventory* (Frisch et al., 2005). MIST-A is a previously validated self-report questionnaire that captures features of hair pulling. It contains 15 questions each rated on a scale from 0 (not true for any of the person’s hair pulling) through to 9 (true for all of my hair pulling) (Flessner et al., 2008a). By convention, it yields two scores, corresponding to ‘automatic’ and ‘focused’ hair pulling, which in prior work were used as the basis for four subtypes (high-high, low-high, high-low, high-high) (Flessner et al., 2008a).

Participants also completed the Stop-Signal Task (SST) (Aron et al., 2007) to measure motor inhibitory control, and the Intra-Dimensional/Extra-Dimensional Set-Shifting Task (IDED) (Owen et al., 1991) to measure extra-dimensional (ED) set shifting (flexible responding). The outcome measures were stop-signal reaction time and total errors for the ED shift stage. We included these domains because they have been implicated in trichotillomania (Chamberlain et al., 2006) and related obsessive-compulsive spectrum disorders (Chamberlain et al., 2021).

### 2.3 Data analysis

K-means clustering was used to identify candidate subtypes of TTM based on automatic and focused scores from the MIST. K-means clustering is a data-driven statistical approach that fits a number of centroids and then assigns each datapoint (here, represented by an individual’s MIST-A scores) to the best fitting cluster. Put differently, the method attempts to fit a set of data to a number of distinct clusters that are separated across multidimensional space.

We first fitted k-means models stepwise from 1 cluster upwards, to examine whether a cluster solution would converge, using Cubic Clustering Criterion (CCC) values. Then, based on the prior literature indicating there may be four subtypes of trichotillomania based on the MIST (low-low, low-high, high-low, high-high) (Flessner et al., 2008a), we used k-means clustering to force fit four clusters based on the MISTA automatic and focused subscores. We then compared these putative subtypes on demographic, clinical, and cognitive measures. Groups were compared on measures of interest using analysis of variance (ANOVA) with follow-up t-tests as appropriate for continuous data; or likelihood ratio tests with follow-up post hoc tests as appropriate for categorical data. This being an exploratory study, and in view of the sample size, statistical significance was defined as p<0.05 uncorrected. All analyses were conducted using JMP Pro software. For k-means modelling, while there is no hard rule regarding required sample size, it is generally recommended that sample size is at least 70 times the number of variables (Dolnicar et al., 2014). Here, with two variables (automatic and focused subscores), the recommended sample size was >140 subjects.

## 3 Results

The sample consisted of 238 adults with trichotillomania (mean age=30.1 [SD 8.2] years; 91.6% females).

When k-means cluster models were then fitted iteratively, CCC values were found to be negative and decreasing as the number of clusters increased from 1 upwards, indicating that the structure of the data did not converge on a cluster solution. This indicates that MIST automatic and focused subscores could not identify subtypes of TTM, within this statistical framework, using the CCC values.

Based on prior literature (Flessner et al., 2008a), we then force-fitted a four cluster solution using K-means clustering, which yielded groups hereafter referred to as: low focused-low automatic, low focused-high automatic, high focused-low automatic, and high focused-high automatic pulling (Table 1).

Of the 238 adults, 71 (29.8%) were classified as low focused-low automatic, 67 (28.2%) low focused-high automatic, 38 (16.0%) high focused-low automatic, and 62 (26.1%) high focused-high automatic.

The demographic and clinical characteristics of the groups are presented in Table 2, along with results from the two cognitive measures. The subtypes did not differ significantly for age, race/ethnicity, sex, education levels, age of symptom onset, duration of illness, or cognitive functioning. However, significant differences were found across subtypes for symptom severity on the MGH-HPS and – to a lesser extent – quality of life. The high-focused high-automatic subtype had significantly worse symptom severity than each other subtype. Interestingly, the high-focused low-automatic subtype had significantly lower quality of life than the low-focused high-automatic subtype.

Table 3 shows the number and percentages of people in each subtype with comorbidities. The subtypes did not differ significantly in terms of rates of OCD, PTSD, psychosis, panic disorder, bipolar disorder, body dysmorphic disorder, ADHD, personality disorder, or alcohol / substance use disorders. Depression was more common in the low-focused low-automatic and high-focused low-automatic subtypes, as compared to the low-focused high-automatic and high-focused high-automatic subtypes. Anxiety was more common in the high-focused low-automatic subtype than the other subtypes. Four cases of eating disorder were found in the low-automatic low-focused subtype, with none in the other subtypes.

## 4 Discussion

This study found that the style of hair pulling (i.e. automatic and focused) had some associations with clinical variables, but that trichotillomania did not form coherent subtypes based on these variables when k-means clustering was used. In fact, high scores of both automatic and focused pulling was associated with more severe symptoms of trichotillomania. This is only somewhat consistent with the Flessner and colleagues study (Flessner et al., 2008b) that reported high scores on either automatic or focused pulling were associated with more severe symptoms. The current study also found that a different style of hair pulling (high focused and low automatic pulling) was associated with worse quality of life, more anxiety, and more depression (although depression was also associated with low focused and low automatic pulling). These findings suggest that different styles may have variable associations with different aspects of trichotillomania.

If both high focused and high automatic pulling are associated with greater symptom severity, then these findings may highlight why many people do not respond to most available psychotherapy treatments. There is some indication that habit reversal therapy (which has been incorporated to some degree in most therapies for trichotillomania) works best for focused pulling (McGuire et al., 2020), but pulling may be driven by both high focused and high automatic styles, not just one. Therefore, one could imagine that a therapy to ameliorate the automatic behavior, combined with habit reversal therapy for the focused style, would be optimal to reduce symptom severity. Alternatively, perhaps strengthening/extending the ‘awareness training’ component of habit reversal therapy could aid its ability to target automatic pulling in conjunction with focused pulling.

The other finding from this study is that the idea of automatic and focused pulling may not be particularly helpful in subtyping trichotillomania. We found that the k-means clustering modelling did not converge on an optimal solution, despite the large sample size in this study, indicating that while automatic and focused hair pulling may have some clinical relevance, there were not any coherent subtypes of the disorder based on these variables. This would be in keeping with the notion that automatic and focused pulling commonly co-occur in the same individual. When we force fitted four subtypes, the findings were not coherent. Collectively, this may be unsurprising as both focused and automatic pulling may characterize the same pulling episode, or certainly the same person across episodes (Christenson et al., 1991, 1993); and therefore not be useful for subtyping.

Potential advantages of this study involve the use of a data-driven clustering methodology, and the relatively large sample size; however, the study has some notable limitations. First, these results are based on secondary analysis of archival data originally collected for other purposes. Second, treatment response was not available and so we cannot evaluate the relationship between automatic and focused pulling and treatment response. Third, we considered automatic and focused pulling scores from the MIST-A, since our studies collected these total scores in particular. Future work could examine other operationalisations of the full MIST such as more recent analysis suggesting Intention and Emotion subscores (Keuthen et al., 2015). Fourth, there are of course other methods that could be used to identify potential subtypes; but our approach has the advantage of being relatively scientifically neutral as it was data-driven.

There is no current evidence that the four proposed types of automatic and focused pulling have distinct neurobiological underpinnings either and thus we are left with multiple and somewhat conflicting reports as to the relevance importance of pulling style in maintaining the behavior. We propose that studies into trichotillomania need to revisit whether subtypes based on focused vs automatic hair pulling have statistical validity and clinical utility. To properly define reproducible subtypes, it is likely going to be necessary to capture a comprehensive range of measurement domains at large sample size. Some initial work has been conducted in efforts to address this (Grant et al., 2020). However, clarification of subtypes is likely to require longitudinal data collection since subtypes may only be apparent over time. It would also be interesting to explore subtypes in terms of response to different treatment modalities for trichotillomania.

## Figures and Tables

**Table 1:** K-means clustering analysis of MIST-A Responses

Cluster	MIST-A Focused	MIST-A Automatic
1	29.7746479	20.8169014
2	31.6865672	32.7761194
3	52.6578947	14.1315789
4	59.5967742	31.2580645

Cluster 1 = low focused-low automatic

Cluster 2 = low focused-high automatic

Cluster 3 = high focused-low automatic

Cluster 4 = high focused-high automatic pulling

**Table 2:** Demographic and clinical characteristics of the sample

								
		1		2		3		4		Statisti c	df	Signific ance (p-value)	Pairwise comparisons
		n	M (SD)	n	M (SD)	n	M (SD)	n	M (SD)				
Age (years)		71	29.4 (7.7)	6 7	30.3 (7.4)	3 8	30.5 (8.9)	62	30.4 (9.4)	0.251f	3, 234	n.s.	
Race/Ethni city [%]	Caucasian	68	[95.8]	5 4	[80.6]	3 0	[79.0]	48	[77.4]	15.79 8c	12	n.s.	
	Mixed (African American and white Caucasian)	0	[0]	0	[0]	0	[0]	0	[0]				
	African American	1	[1.4]	7	[10.4]	3	[7.9]	7	[11.3]				
	Latino/Hispa nic	1	[1.4]	1	[1.5]	1	[2.6]	2	[3.2]				
	Asian	1	[1.4]	3	[4.5]	3	[7.9]	4	[6.5]				
	Other	0	[0]	2	[3.0]	1	[2.6]	1	[1.6]				
Sex [%]	Female	64	[90.1]	5 7	[85.1]	3 7	[97.4]	60	[96.8]	10.42 1c	6	n.s.	
	Male	6	[8.5]	1 0	[14.9]	1	[2.6]	2	[3.2]				
	Intersex	1	[1.4]	0	[0]	0	[0]	0	[0]				
Education		41	3.9 (1.0)	2 7	3.7 (1.4)	2 1	3.6 (1.2)	22	3.4 (1.4)	0.783f	3, 107	n.s.	
Age of Onset		14	12.4 (3.7)	1 5	14.8 (3.7)	1 6	12.1 (9.6)	19	12.6 (9.4)	0.412f	3, 60	n.s.	
Duration of Illness (years)		14	20.6 (8.5)	1 5	15.9 (9.5)	1 6	17.9 (7.5)	19	18.8 (15.8)	0.434f	3, 60	n.s.	
MGH-HPS Total		70	17.1 (3.8)	6 6	17.7 (3.7)	3 8	16.6 (4.5)	62	19.5 (3.6)	6.198f	3, 232	0.0005 **	**4 vs. 3** ** **4 vs. 1** ** **4 vs. 2** **
QOL T-score		41	44.0 (13.0)	2 6	48.5 (11.3)	2 0	37.7 (11.6)	22	42.4 (11.2)	3.195f	3, 97	0.027*	2 vs. 3**
IED Errors (block 8)		41	11.3 (10.6)	2 4	10.6 (10.5)	1 9	10.8 (11.7)	17	9.9 (9.8)	0.071f	3, 97	n.s.	
SST SSRT (last half)		41	211.8 (51.9)	2 4	210.3 (122.8)	1 9	194.3 (74.0)	15	203.8 (92.5)	0.208f	3, 95	n.s.	16

**1**= Low focused, low automatic; **2**= Low focused, high automatic; **3**= High focused, low automatic; **4**= High focused, high automatic (according to MIST-A: Milwaukee Inventory for Subtypes of Trichotillomania-Adults)

All results are mean (SD) unless otherwise noted

Statistic: c= Chi-square; f= F ratio

Bold p-value indicates significance at *p < 0.05, **p < 0.01 with effect size; n.s.= not significant

Abbreviations: MGH-HPS= Massachusetts General Hospital Hairpulling Scale; QOL= The Quality of Life Questionnaire; IED= Intra-Extra Dimensional Set Shift; SST SSRT= Stop Signal Task – Stop Signal Reaction Time

**Table 3:** Psychiatric Conditions in Participants Grouped by MIST-A Cluster

		Cluster 1		Cluster 2		Cluster 3		Cluster 4					
										Statistic	df	Significance (p-value)	Pairwise comparisons
Depression	No	52	73.2	61	91.0	29	76.3	56	90.3	11.626	3	0.009**	2 vs. 1** 3 vs. 2* 4 vs. 1*
	Yes	19	26.8	6	9.0	9	23.7	6	9.7				
Anxiety	No	62	87.3	62	92.5	28	73.7	57	91.9	8.283	3	0.041*	3 vs. 2* 4 vs. 3*
	Yes	9	12.7	5	7.5	10	26.3	5	8.1				
OCD	No	70	98.6	65	97.0	36	94.7	60	96.8	1.323	3	n.s.	
	Yes	1	1.4	2	3.0	2	5.3	2	3.2				
PTSD	No	68	95.8	66	98.5	37	97.4	61	98.4	1.277	3	n.s.	
	Yes	3	4.2	1	1.5	1	2.6	1	1.6				
Psychotic Disorder	No	44	100	55	100	33	100	59	100	0.000	3	-	
	Yes	0	0	0	0	0	0	0	0				
Panic Disorder	No	69	97.2	66	98.5	37	100	62	100	3.563	3	n.s.	
	Yes	2	2.8	1	1.5	0	0	0	0				
Bipolar	No	70	100	67	100	38	100	62	100	0.000	3	-	
	Yes	0	0	0	0	0	0	0	0				
Body Dysmorphic Disorder	No	69	98.6	67	100	38	100	62	100	2.449	3	n.s.	
	Yes	1	1.4	0	0	0	0	0	0				
ADD/ADHD	No	13	92.9	14	93.3	16	100	15	79.0	5.715	3	n.s.	
	Yes	1	7.1	1	6.7	0	0	4	21.0				
Personality Disorder	No	14	100	15	100	15	93.8	19	100	2.821	3	n.s.	
	Yes	0	0	0	0	1	6.2	0	0				
AUD/SUD	No	70	98.6	67	100	38	100	61	100	2.421	3	n.s.	
	Yes	1	1.4	0	0	0	0	0	0				
Eating Disorder	No	67	94.4	67	100	38	100	61	100	9.805	3	0.020*	**4 cases in the low-focused low-automatic (post hoc tests not done due to low cell count)**
	Yes	4	5.6	0	0	0	0	0	0				

**1**= Low focused, low automatic; **2**= Low focused, high automatic; **3**= High focused, low automatic; **4**= High focused, high automatic (according to MIST-A: Milwaukee Inventory for Subtypes of Trichotillomania-Adults)

All values are n (%) unless stated otherwise

Bold p-value indicates significance at *p < 0.05, **p < 0.01 with effect size; n.s.= not significant

Abbreviations: ADD= Attention deficit disorder; ADHD= Attention deficit hyperactivity disorder; AUD= Alcohol use disorder; SUD= Substance use disorder
